# Laparoscopic cholecystectomy in the treatment of acute cholecystitis: comparison of results between early and late cholecystectomy

**DOI:** 10.11604/pamj.2017.26.49.8359

**Published:** 2017-01-31

**Authors:** Turan Acar, Erdinç Kamer, Nihan Acar, Kemal Atahan, Halis Bağ, Mehmet Hacıyanlı, Özgün Akgül

**Affiliations:** 1 General Surgery, MD, Ỉzmir Katip Çelebi University Atatürk Training and Research Hospital General Surgery Clinic, Turkey

**Keywords:** Laparoscopic cholecystectomy, acute cholecystitis, early, late, morbidite

## Abstract

**Introduction:**

Laparoscopic cholecystectomy has become the gold standard in the treatment of symptomatic gallstones. The common opinion about treatment of acute cholecystitis is initially conservative treatment due to preventing complications of inflamation and following laparoscopic cholecystectomy after 6- 8 weeks. However with the increase of laparoscopic experience in recent years, early laparoscopic cholecystectomy has become more common.

**Methods:**

We aimed to compare the outcomes of the patients to whom we applied early or late cholecystectomy after hospitalization from the emergency department with the diagnosis of AC between March 2012-2015.

**Results:**

We retrospectively reviewed the files of totally 66 patients in whom we performed early cholecystectomy (within the first 24 hours) (n: 33) and to whom we firstly administered conservative therapy and performed late cholecystectomy (after 6 to 8 weeks) (n: 33) after hospitalization from the emergency department with the diagnosis of acute cholecystitis. The groups were made up of patients who had similar clinical and demographic characteristics. While there were no statistically significant differences between the durations of operation, the durations of hospitalization were longer in those who underwent early cholecystectomy. Moreover, more complications were seen in the patients who underwent early cholecystectomy although the difference was not statistically significant.

**Conclusion:**

Early cholecystectomy is known to significantly reduce the costs in patients with acute cholecystitis. However, switching to open surgery as well as increase of complications in patients who admitted with severe inflammation attack and who have high comorbidity, caution should be exercised when selecting patients for early operation.

## Introduction

The incidence of gallstones is 10-15% and the lifetime recurrence rate of symptoms or complications in such patients is about 35% [1]. Laparoscopic cholecystectomy has become the gold standard in the treatment of symptomatic gallstones. The major advantages of laparoscopic cholecystectomy (LC) include less postoperative pain, less time required for hospitalization and recovery, and better cosmetic results [2]. The general view in the treatment of acute cholecystitis (AC) is to firstly administer conservative therapy to prevent possible complications associated with inflammation and then after 6 to 8 weeks, to perform laparoscopic cholecystectomy [3]. Although over 70% of such patients respond to medical therapy within the first 24 to 48 hours, LC is the definitive treatment method for treatment of symptomatic gallstone disease. In the past, open surgery was recommended considering the complications associated with operation and prolonged hospitalization [4]; moreover, performing emergency LC was difficult most of the times because of the patient comorbidity and the difficulty of availability of appropriate equipment and surgery room conditions in emergency cases. However, the recently increasing laparoscopic experience and the favorable results of the meta-analyses published on this prompts surgeons to perform early LC intervention [5, 6].

## Methods

We aimed to compare the outcomes of the patients to whom we applied early or late cholecystectomy after hospitalization from the emergency department with the diagnosis of AC between March 2012 and March 2015. AC diagnosis was made according to the Tokyo criteria, after reviewing the physical examination, laboratory and imaging methods. The patients were divided into two groups and their files were reviewed retrospectively. The groups were made up of patients who had similar clinical and demographic characteristics. We included the patients in whom we performed operation within the first 24 hours after hospitalization from the emergency department in the early cholecystectomy group and those to whom we firstly administered medical therapy (intravenous antibiotics, fluid and analgesics) and operated after 6 to 8 weeks in the late cholecystectomy group. Second generation cephalosporins and metronidazole were used as the medical therapy. Surgical interventions were made by totally six different surgeons as two groups of three who had identical experience.

We compared the clinical and demographic characteristics, physical examinations, laboratory results and radiological, intraoperative and postoperative data between the groups. We tried to create a scale by detailing the causes of morbidity and the reasons for switching to open surgery. Pearson’s Chi-Square, Fisher´s Exact test, Mann-Whitney U test (distribution was not normal. Kolmogorov-Smirnov and Shapiro-Wilks p < 0.05) statistical tests were used. Written informed consents were obtained from the patients after giving detailed information to them about their disease and the procedures to be performed and have been performed. The consents required to perform a scientific study were obtained from the patients.

## Results

We retrospectively reviewed the files of totally 66 patients in whom we performed early cholecystectomy (within the first 24 hours) (n: 33) and to whom we firstly administered conservative therapy and performed late cholecystectomy (after 6 to 8 weeks) (n: 33) after hospitalization from the emergency department with the diagnosis of acute cholecystitis. The physical examination findings were similar in both groups. Tenderness had been found in the abdominal examinations of all patients. The clinical and demographic characteristics of the patients are shown in Table 1, the physical, laboratory and radiological data in Table 2 and intraoperative and postoperative data in Table 3. We found statistically significant differences between the two groups in patients’ ages, previous operation history and comorbidity, body temperature, defense, rebound and Murphy positivity at admission as well as presence of pericholecystic fluid (p < 0.05). These parameters comprised the majority of the patients in whom early switching to open surgery occurred or complications developed. While there were no statistically significant differences between the durations of operation, the durations of hospitalization were longer in those who underwent early cholecystectomy (p: 0.006). Moreover, more complications were seen in the patients who underwent early cholecystectomy although the difference was not statistically significant (P: 0.105) (Table 4). The main reasons for switching to open surgery are shown in Table 5. The majority of these are composed of the difficulties of anatomy. The rates of switching to open surgery were higher in the group who underwent early LC (p: 0.020).

**Table 1:** Clinical and demographic characteristics of the patients who underwent early and late cholecystectomy

	Group 1 LC within 24 h (n = 33)	Group 2 LC after 24 h (n = 33)	p
Age	63.45±16.55	53.67±13.19	0.029
Gender, n (%) Male Female	17 (51.5)	11 (33.3)	0.135
	16 (48.5)	22 (66.7)	
Mean pain duration (days)	3.49±1.52	3.18±1.21	0.466
Number of patients who experienced two or more attacks, n (%)	20 (60.6)	9 (27.3)	0.006
ERCP history, n (%)	7 (21.2)	9 (27.3)	0.566
Previous abdominal operation, n (%)	16 (48.5)	9 (27.3)	0.076
Body temperature (mean), C	38.21±0.73	37.8±0.55	0.021
Comorbidity, n (%)	26 (78.8)	16 (48.5)	0.011

**Table 2:** Physical, laboratory and radiological data of the patients who underwent early and late cholecystectomy

	Group 1LC within 24 h(n = 33)	Group 2LC after 24h (n = 33)	p
**Physical exam findings, n (%)**			
Tenderness	33 (100)	33 (100)	-
Defense	29 (87.9)	20 (60.6)	0.011
Rebound	20 (60.6)	2 (6.1)	0.000
Murphy	23 (69.7)	3 (9.1)	0.000
**Laboratory (mean)**			
WBC (4*10^9^ /L- 11* 10^9^ /L)	14.55±6.95	11.34±3.18	0.088
ALT (0- 41 U/L)	80.7±97.27	125.64±131.94	0.086
AST (0- 40 U/L)	76.82±88.16	108.79±109.41	0.067
Amylase (28- 100 U/L)	372.09±929.72	243.73±536.08	0.581
Total bilirubin (< 1,4 mg/ dL)	1.74±1.61	1.46±1.03	0.773
Direct bilirubin (< 0,2 mg/ dL)	0.89±0.88	0.88±0.58	0.483
**Ultrasonography findings (%)**			
Gallstone, n (%)	33 (%100)	33 (%100)	-
Increased wall thickness of gallbladder, n (%)	27 (81.8)	31 (93.9)	0.258
Pericholecystic fluid, n (%)	18 (54.5)	6 (18.2)	0.002
Biliary tract dilation, n (%)	4 (12.1)	5 (15.2)	1.000

**Table 3:** Intraoperative and postoperative data of the patients who underwent early and late cholecystectomy

	Group 1LC within 24 h(n = 33)	Group 2LC after 24 h(n = 33)	p
Duration of operation (minutes)	95.36±32.9	95.73±22.94	0.529
Duration of hospitalization (days)	5.03±4.91	2.36±1.95	0.006
Intraoperative or postoperative complication, n (%) (Morbidity)	6 (18.2)	1 (3)	0.105
Rate of switching to open surgery, n (%)	16 (48.5)	7 (21.2)	0.020

**Table 4:** Causes of morbidity

Complications	Group 1LC within 24 h(n = 33)	Group 2LC after 24 h(n = 33)	p
Wound infection, n (%)	2 (6.1%)		
Biliary leakage, n (%)	2 (6.1%)	1 (3%)	
Bleeding, n (%)	1 (3%)		
Bile duct injury, n (%)	1 (3%)		
Total, n (%)	6 (18.2)	1 (3)	0.105

**Table 5:** Reasons for switching to open surgery

Conversion, n (%)	Group 1LC within 24 h(n = 33)	Group 2LC after 24 h(n = 33)	p
Difficulty identifying anatomy	8 (24.3%)	4 (12.1%)	
Adhesions	4 (12.1%)	2 (6.1%)	
Haemorrhage	3 (9.1%)	1 (3%)	
Biliary tract injury	1 (3%)	-	
Total	16 (48.5)	7 (21.2)	0.020

## Discussion

The mean age of patients presenting with acute cholecystitis varies between 40 and 80 years. This condition is seen three times more in women than men. Acute inflammation should be suspected in patients with gallstones in the presence of pain in upper right quadrant which continues despite analgesia. History includes occasional biliary colic attacks. Physical examinations generally reveal moderate fever, tachycardia and marked tenderness in the upper right quadrant. 25% of patients have palpable hydropic gallbladder. The classical Murphy symptom, which is characterized by the abrupt arrest of breathing when a direct palpation is applied onto the gallbladder. Laboratory values may reveal leukocytosis and mildly increased levels of bilirubin, alkaline phosphatase, transaminase and amylase. Ultrasonography is highly important in the diagnosis. Presence of gallstones or gallbladder sludge, gallbladder wall thickening of 4 mm or above, and detection of pericholecystic fluid support the diagnosis of acute cholecystitis. The Tokyo criteria were defined for the diagnosis and determination of the severity of the disease [7]. According to these criteria, patients who show Murphy’s sign, any local symptoms such as mass, pain or tenderness in the right hypochondrium, and any systemic symptoms such as fever, leukocytosis or increased C-reactive protein are diagnosed with AC. This report also classifies AC based on the severity. According to this, in mild cholecystitis (grade 1) the inflammation is limited to the gallbladder and no organ dysfunction is seen. In moderate cholecystitis (grade 2) no organ dysfunction but advanced inflammation is seen. In severe cholecystitis (grade 3) organ dysfunction is present. According to this classification, is is stated that percutaneous transhepatic bile drainage (PTHBD) may be preferred in the patient group who have high comorbidity and moderate or severe AC [8]. It is emphasized that a marked clinical improvement and a marked reduction in morbidity can be observed in this way [9, 10].

It is a standard practice to administer fluid, analgesia and broad-spectrum antibiotics in the initial treatment of acute cholecystitis. During the first years of laparoscopic surgery, LC was recognized as a relative contraindication in AC. But especially during the recent years, LC has been demonstrated to be safe in the treatment of AC [11]. However, the appropriate timing for this procedure is still a question of debate. In some studies, it is considered that the optimal timing for LC is 6 to 9 weeks after the conservative therapy taking into consideration the general condition an comorbidities of the patients and projecting that the acute inflammation will resolve in 6 weeks [12–14].

However, those who advocate early LC state that in patients who are prepared for late LC after the conservative therapy, the fibrotic adhesions developed between the gallbladder and the surrounding structures following inflammation and edema may cause excessively difficult dissection [15]. They advocate that, early LC performed with appropriate timing in correctly selected patients will facilitate the dissection of edematous plane, and reduce morbidity as well as the possibility of late complications such as emphysematous or gangrenous cholecystitis [16].

Biliary tract injury is one of the most frightening complications that may develop during LC. It can even be fatal due to sepsis. Even the corrective surgical procedures can lead to high morbidity and mortality rates and impair quality of life [17, 18]. The increasing experience, improved skills and new tools have reduced especially biliary tract injuries and associated morbidities in early LC [19–22]. A meta-analysis conducted by Japanese researchers demonstrated that early laparoscopic cholecystectomy performed within 24 to 96 hours in acute cholecystitis ensure shorter hospitalization while having similar complication rates compared to late laparoscopic cholecystectomy [9].

Condilis et al. divided their patients into three groups: Those they performed operation during the first 48 hours (group 1), after 48 hours to 4 weeks (group 2) and after 5 to 8 weeks (group 3) [23]. The highest rates of switching to open surgery were seen in group 2, and the highest rates of complications and postoperative hospitalization wer seen in group 1.

In the studies conducted by Popkharitov et al. and Lee et al. [24, 25] the patients were again divided into three groups. They classified the patients they operated within the first 72 hours as those who early operated. Neither study demonstrated a significant difference between the groups in terms of switching to open surgery, complications or duration of hospitalization. The study conducted by Masayuki et al. did not show differences between the groups in terms of switching to open surgery, duration of operation, blood loss, postoperative morbidity or duration of hospitalization either [26]. In all three studies, it was suggested that LC should be performed at admission in patients with acute cholecystitis. In our study, more complications were observed, although not statistically significant in patients in whom early cholecystectomy was performed [18.2% - 3%]. We attributed the increased complication rates in the early LC compared to the literature to wrong patient selection and insufficient experience.

When the studies performed are reviewed, it is seen that the rates of switching to open surgery are considerably different [0-39%] [21, 27, 28]. The reason of this considerable difference can be attributed to the different demographics of the patients, the severity of inflammation, the experience of the surgeons and the timing. The study conducted by Abdulmohsen et al. found a rate of 6.7% and the authors stated that their center has a great experience in gallbladder surgery [29]. We found these rates as 16% is early surgery and 7% in late surgery. A statistically significant difference was found between the two groups [P: 0.020]. In our study, the most common reason for switching to open surgery was the fact that Callot’s dissection could not be properly performed due to anatomical difficulty associated with inflammation.

## Conclusion

Early cholecystectomy is known to significantly reduce the costs in patients with acute cholecystitis. However, given the facts that prolongation of hospitalization and switching to open surgery as well as increase of complications in patients who admitted with severe inflammation attack and who have high comorbidity, caution should be exercised when selecting patients for early operation. Each clinic should consider the patient and physician factors, the population and the type of medical facility when making a decision with respect to the timing of the operation. Maybe a new treatment scale containing these data should be created for the treatment of acute cholecystitis.

### What is known about this topic

- The Tokyo criteria were defined for the diagnosis and determination of the severity of the disease;
- It is advocated that, early LC performed with appropriate timing in correctly selected patients will facilitate the dissection of edematous plane, and reduce morbidity as well as the possibility of late complications such as emphysematous or gangrenous cholecystitis.

### What this study adds

- Each clinic should consider the patient and physician factors, the population and the type of medical facility when making a decision with respect to the timing of the operation.
